# Long-term risk of venous thromboembolism after COVID-19 mainly related to intensive care unit stay based on findings of a self-controlled case series study

**DOI:** 10.1016/j.rpth.2023.102210

**Published:** 2023-09-29

**Authors:** Ioannis Katsoularis

**Affiliations:** Department of Public Health and Clinical Medicine, Umeå University, Umeå, Sweden

To the Editor,

Thank you for a very interesting study by Sjöland et al. [1]. This study is a population-based register cohort study from Sweden investigating the long-term risk of venous thromboembolism (VTE) during days 60 to 180 and >180 days following COVID-19. The authors of the study showed that the long-term risk of VTE, and particularly pulmonary embolism (PE), was increased following the infection and was highest among individuals hospitalized for COVID-19. We have previously published a study investigating the risk of VTE and bleeding following COVID-19 [2]. The 2 studies had similar study populations, study periods, and data sources and showed comparable results. However, different study methodologies were used, which led to some substantial differences in the results that should be acknowledged.

The study by Sjöland et al. [1] was a cohort study, while our study [2] had a dual methodological approach. Two complementary study methodologies were used to increase the confidence in the validity of our results: a cohort study design and a self-controlled case series (SCCS) method. The SCCS is a modern study design that compares intraperson incidence rates of an outcome in different time periods relative to exposure based on the relative timing of the outcome events within the observation period [3]. Individuals act as their own controls, and the analyses are automatically adjusted for the effect of time-fixed (measured or unmeasured) confounders, reducing the risk of bias and residual confounding [3]. In addition, we performed a stratification analysis based on disease severity, where stay in the intensive care unit (ICU) was used as a separate subgroup.

Among individuals with COVID-19 without hospitalization, the 2 studies found approximately the same duration of excess risk of VTE, which was normalized after the second month following the infection. Sjöland et al. found a borderline statistically significant excess risk in the period 60 to 180 days (hazard ratio, 1.17; 95% CI, 1.01-1.35) and not any elevated risk during the period >180 days following the infection.

Sjöland et al. showed that the risk of PE and deep vein thrombosis among hospitalized individuals with COVID-19 remained increased even during the period 60 to 180 days. However, in the stratification analyses of the SCCS study we performed, we could distinguish that this excess risk was mainly related to ICU stay. For individuals hospitalized but not admitted to the ICU, the risk of deep vein thrombosis and PE returned to baseline after days 60 and 90, respectively, in our study.

Interestingly, Sjöland et al. also showed that the excess risk of VTE in hospitalized individuals remained elevated beyond 180 days after the infection. This might be the case only for individuals with COVID-19 admitted to the ICU, while for individuals hospitalized but not admitted to the ICU, the risk of VTE in our study returned to baseline before day 180 following the infection. Moreover, as we did not include any postexposure risk period after day 180 in our SCCS studies, our results might have been biased to the null, leading to underestimation of the risks reported.

The results of the 2 studies are comparable. Nevertheless, due to differences in study methodology, apparently the use of the SCCS method in our study and the consideration of individuals admitted to the ICU as a separate group, we could show that the long-term risk of VTE after COVID-19 was mainly related to individuals admitted to the ICU.

On behalf of the authors of the study by Katsoularis et al. [2].

Best regards,

Ioannis Katsoularis, M.D.,

Department of Public Health and Clinical Medicine, Umeå University

## References

[1] Sjoland H., Lindgren M., Toska T., Hansson P.O., Sandblad K.G., Alex C. (2023). Pulmonary embolism and deep venous thrombosis after COVID-19: long-term risk in a population-based cohort study. Res Pract Thromb Haemost.

[2] Katsoularis I., Fonseca-Rodríguez O., Farrington P., Jerndal H., Lundevaller E.H., Sund M. (2022). Risks of deep vein thrombosis, pulmonary embolism, and bleeding after covid-19: nationwide self-controlled cases series and matched cohort study. BMJ.

[3] Whitaker H.J., Farrington C.P., Spiessens B., Musonda P. (2006). Tutorial in biostatistics: the self-controlled case series method. Stat Med.

